# Supplementary material for: Improving Geometric Validation Metrics and Ensuring Consistency with Experimental Data through TrioSA: An NMR Refinement Protocol
Source: Int J Mol Sci. 2023 Aug 28;24(17):13337. doi: 10.3390/ijms241713337 (PMC10487420; doi:10.3390/ijms241713337)
Supplement: Supplementary file 1 [file ijms-24-13337-s001.zip › Supplementary_file1_Supplementary information.pdf]

Supplementary Material

Improving Geometric Validation Metrics and Ensuring Consistency with experimental data through TrioSA: An NMR Refinement Protocol

Article type: ORIGINAL RESEARCH

Authors and affiliations:

Youngbeom Cho∗†,‡ Hyojung Ryu ,† Gyutae Lim,† Seungyoon Nam §,¶ Jinhyuk Lee §†‡

∗First author
§Correponding author
†Department of Bioinformatics, KRIBB School of Bioscience, University of Science and Technology (UST), Daejeon 34141, Republic of Korea
‡Disease Target Structure Research Center, Korea Research Institute of Bioscience and Biotechnology (KRIBB), Daejeon 34141, Republic of Korea
¶Department of Genome Medicine and Science, AI Convergence Center for Medical Science, Gachon Institute of Genome Medicine and Science, Gachon University Gil Medical Center, Gachon University College of Medicine, Incheon, 21565, Korea; Department of Health Sciences and Technology, Gachon Advanced Institute for Health Sciences and Technology, Gachon University, Incheon 21999, Korea

Corresponding author:

Jinhyuk Lee, Disease Target Structure Research Center, Korea Research Institute of Bioscience and Biotechnology (KRIBB), Daejeon 34141, Korea, jinhyuk@kribb.re.kr

Supplementary information includes:

S1: Supplementary Figures S1-S3
S2: Supplementary Tables S1-S5
S3. Supplementary Excel Tables, provided as Supplementary files 2-6

Figure S1. Comparison of structural quality metrics for five NMR refinement protocols, including Initial, SA, SA+GBSW, SA+STAP, and Refined (SA+GBSW+STAP) from 2017 to 2022. Histograms depict the distribution of (A) normalized DOPE score, (B) Clashscore, (C) Ramachandran score (residues in the allowed region), and (D) Rotamer normality Z-score. These metrics were used to assess the improvement in structural quality of the refinement protocols. Standard deviation bars represent the variability in the data for each refinement protocol.

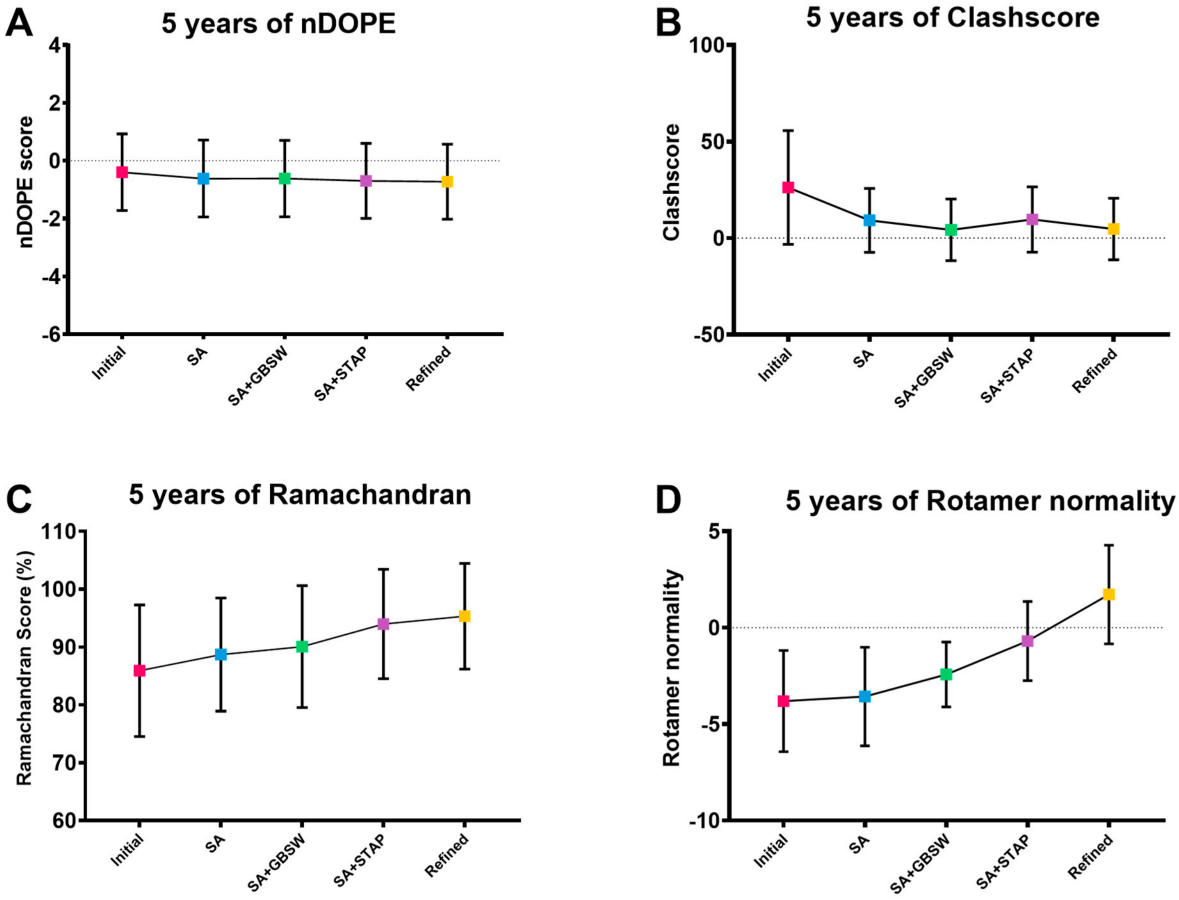

Figure S2. Histograms showing the distribution of structural quality metrics for X-ray and NMR structures from 1976 to 2022. (A) Normalized DOPE score, (B) Clashscore, (C) Ramachandran score (percentage of residues in allowed regions), and (D) Rotamer normality Z-score.

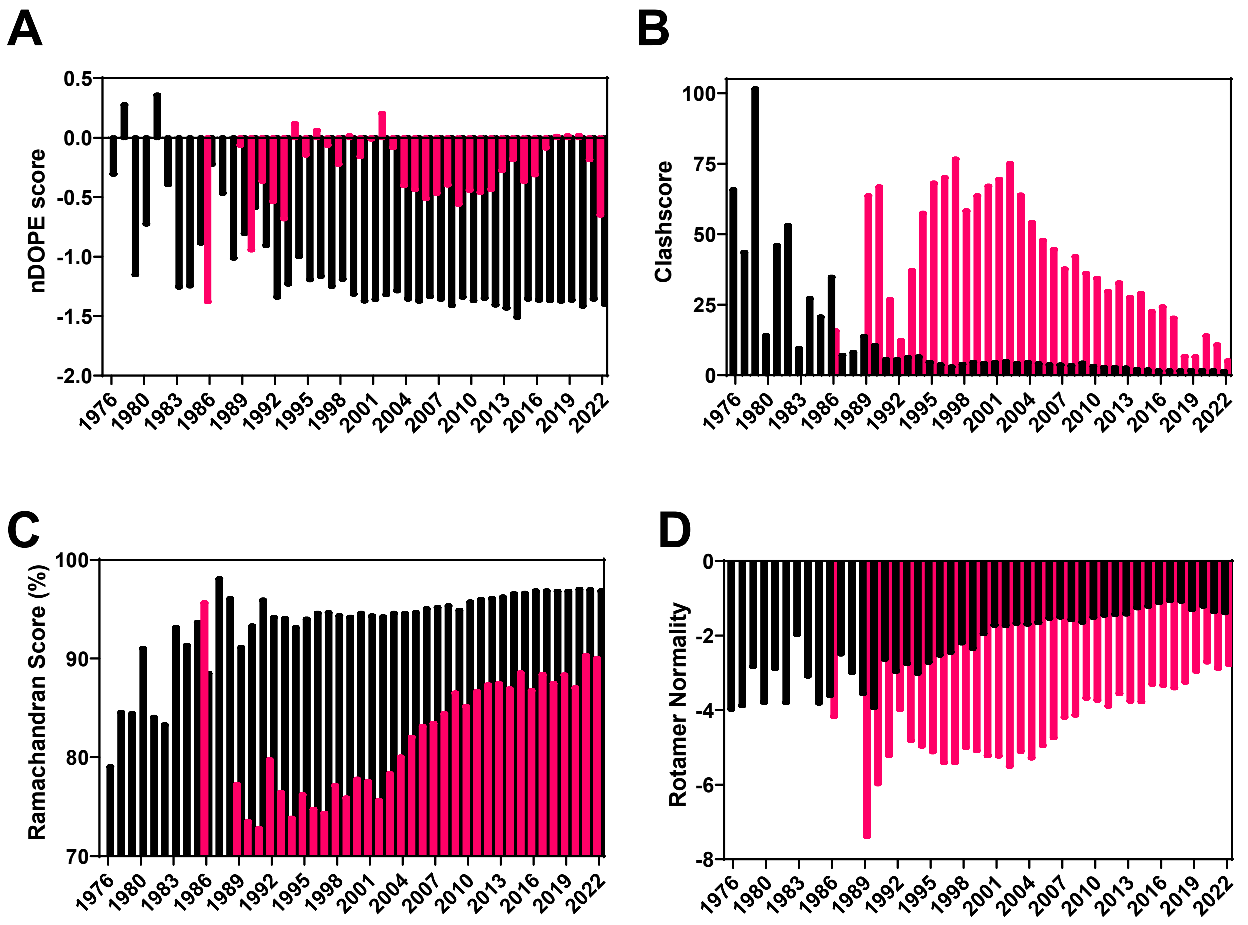

Figure S3 Scatter plot of Geometrical Validation Metrics vs TM-score. (A) shows the scatter plot of nDOPE vs TM-score, separated by the range of TM-score (all, 0.5>, 0.8>). (B) displays the scatter plot of Ramachandran score vs TM-score, separated by the range of TM-score (all, 0.5>, 0.8>). (C) shows the scatter plot of Clashscore vs TM-score, separated by the range of TM-score (all, 0.5>, 0.8>). Finally, (D) presents the scatter plot of Rotamer normality vs TM-score, separated by the range of TM-score (all, 0.5>, 0.8>).

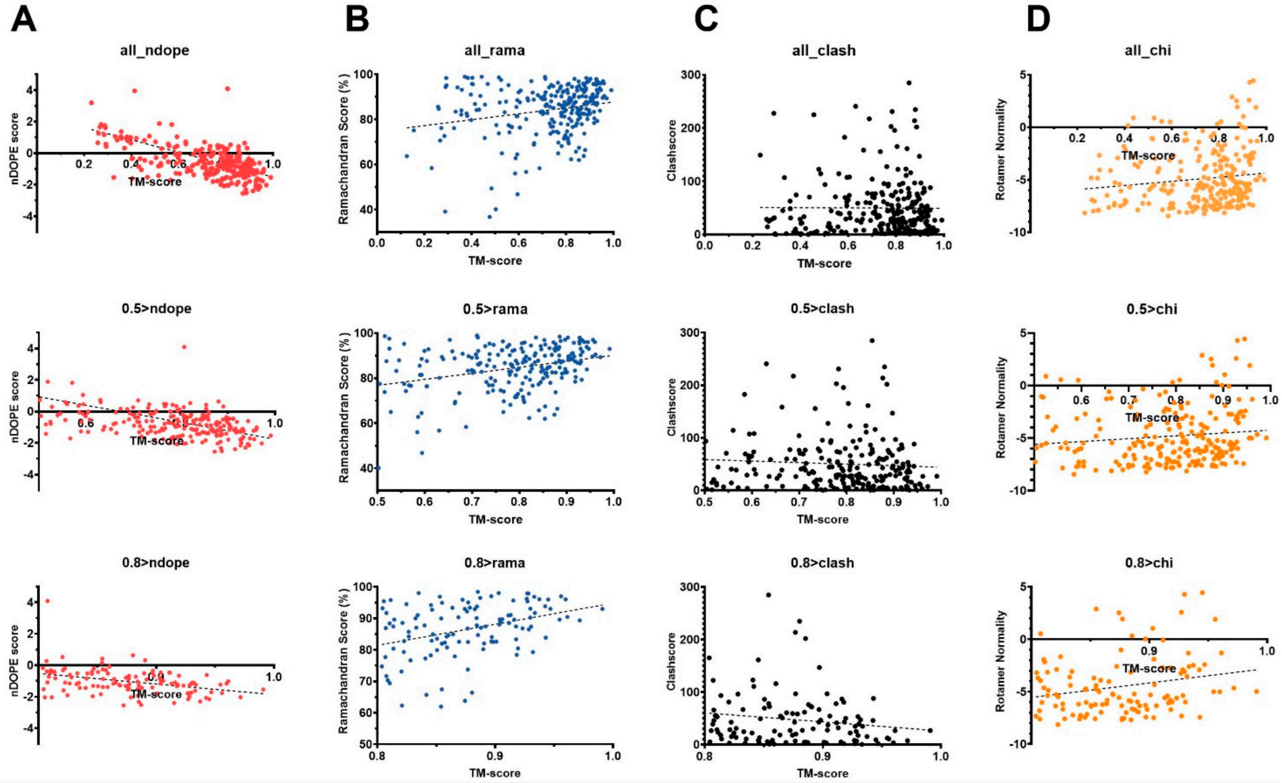

Table S1. Comparative analysis of geometrical validation metrics before and after refinement using TrioSA and RECOORD database.

	nDOPE		Clashscore		Ramachandra score		Rotamer normality	
	Mean	SD	Mean	SD	Mean	SD	Mean	SD
Initial	-0.20	1.01	74.14	61.87	77.06	12.22	-6.21	1.92
Refined	-0.94	1.08	3.09	3.00	95.89	3.81	2.24	1.73
CNS	-0.40	0.93	21.36	15.85	79.32	9.93	-2.35	1.58
CNW	-0.93	0.91	15.50	7.42	81.72	7.54	-2.49	1.11
CYA	-0.17	0.94	59.89	32.93	69.08	11.22	-7.27	0.54
CYW	-0.93	0.91	15.80	7.38	81.08	7.51	-2.78	1.10

Table S2. Validation Assessment of 3,752 Initial and Refined NMR Protein Structures. The Z-score indicates a positive value above the mean average. Using RMS Z-scores, a score of 1 represents an ideal result.

Quality assessment scores			Initial structures	Refined structures
NOE violation	All	Max/RMSD	1.41/0.12	0.71/0.07
		Number of violated NOE (0.0/0.5/1.0/2.0)	113.10/19.39/10.70/3.98	132.89/8.08/2.43/0.69
	Intra	Max/RMSD	0.41/0.05	0.36/0.04
		Number of violated NOE (0.0/0.5/1.0/2.0)	23.24/3.37/1.40/0.47	24.93/2.32/0.87/0.33
	Sequential	Max/RMSD	0.67/0.08	0.51/0.07
		Number of violated NOE (0.0/0.5/1.0/2.0)	26.63/3.96/2.01/0.62	37.43/2.15/0.54/0.12
	Medium	Max/RMSD	0.90/0.14	0.49/0.08
		Number of violated NOE (0.0/0.5/1.0/2.0)	27.54/4.40/2.50/0.85	31.78/1.38/0.32/0.05
	Long	Max/RMSD	1.10/0.18	0.54/0.08
		Number of violated NOE (0.0/0.5/1.0/2.0)	35.68 /7.66/4.78/2.03	38.74/2.23/0.70/0.19
Dihedral angle violation		Max/Ave	28.57/0.68	17.12/0.52
Geometrical validation scores	Optimal protein energy			
	DOPE		-7099.04	-7811.72
	nDOPE		-0.20	-0.94
	dDFIRE		-144.24	-163.98
	Steric clashscore		42.21	3.86
	Ramachandran plot appearance			
	MolProbity		83.66	96.75
	PROCHECK		75.99	92.37
	What_CHECK structure Z-score			
	1st pack		-3.30	-2.60
	2nd pack		-2.59	-1.40
	Ramachandran plot appearance		-4.16	2.84
	Rotamer normality		-4.80	2.31
	Backbone conformation		-1.45	-1.19

Table S3. The summary of predicting protein-ligand docking residues. MCC comparison of 14 pairs of X-ray and NMR protein structures

X-ray pdb id	NMR pdb id	MCC of X-ray	MCC of initial NMR	MCC of refined NMR
1BWO	1GH1	0.761	0.225	0.396
1ES1	1J0Q	0.623	0.148	0.457
1HVH	1BVE	0.784	0.759	0.710
1M2M	1F04	0.721	0.365	0.289
2VBU	2P3M	0.925	0.645	0.652
3AKM	2MJI	0.902	0.435	0.591
3UQI	2LPV	0.703	0.278	0.422
4MNS	6R3C	0.872	0.294	0.476
5FSL	1IRY	0.868	0.263	0.205
7WW5	1MUT	0.694	0.417	0.630
7WW5	1TUM	0.694	0.304	0.399
3ZCF	1J3S	0.634	0.391	0.361
1CTQ	1CRP	0.754	0.774	0.796
5YOK	1Q9P	0.827	0.480	0.555

Table S4. The summary of maximum and average distance nuclear overhauser effect (NOE) RMSD of five NMR refinement protocols. In this analysis, 3,752 NMR protein structures with distance experimental data in BMRB were employed.

	Maximum distance NOE RMSD	Average distance NOE RMSD
Initial	1.41	0.12
SA	0.68	0.07
SA+GBSW	0.61	0.06
SA+STAP	0.77	0.08
TrioSA	0.71	0.07

Table S5. The summary of maximum and average dihedral angle nuclear NOE RMSD of five NMR refinement protocols. In this analysis, 2,318 NMR protein structures with dihedral angle experimental data in BMRB were employed.

	Maximum dihedral angle NOE RMSD	Average dihedral angle NOE RMSD
Initial	28.57	0.68
SA	16.78	0.54
SA+GBSW	14.83	0.47
SA+STAP	17.10	0.51
TrioSA	17.12	0.52

S3: Supplementary Excel Tables, provided as additional files 2-6

3.1 Supplementary file2

The results of identify the appropriate MD steps and temperature

3.2 Supplementary file 3

The validation assessments of five NMR refinement protocols

3.3 Supplementary file 4

The results of analysis of distance NOE using five NMR refinement protocols.

3.4 Supplementary file 5

3,752 of Refined NMR protein structure PDB ID

3.5 Supplementary file 6

Comparative analysis of structural quality metrics for TrioSA and RECOORD database.
